# Selective NLRP3 Inflammasome Inhibitor MCC950 Suppresses Inflammation and Facilitates Healing in Vascular Materials

**DOI:** 10.1002/advs.202300521

**Published:** 2023-05-07

**Authors:** Angus J. Grant, Nianji Yang, Matthew J. Moore, Yuen Ting Lam, Praveesuda L. Michael, Alex H.P. Chan, Miguel Santos, Jelena Rnjak‐Kovacina, Richard P. Tan, Steven G. Wise

**Affiliations:** ^1^ School of Medical Sciences Faculty of Health and Medicine Charles Perkins Centre University of Sydney Sydney NSW 2006 Australia; ^2^ Graduate School of Biomedical Engineering University of New South Wales Sydney NSW 2006 Australia

**Keywords:** MCC950, neointimal hyperplasia, NLRP3, restenosis, vascular inflammation

## Abstract

Minimally invasive interventions using drug‐eluting stents or balloons are a first‐line treatment for certain occlusive cardiovascular diseases, but the major long‐term cause of failure is neointimal hyperplasia (NIH). The drugs eluted from these devices are non‐specific anti‐proliferative drugs, such as paclitaxel (PTX) or sirolimus (SMS), which do not address the underlying inflammation. MCC950 is a selective inhibitor of the NLRP3‐inflammasome, which drives sterile inflammation commonly observed in NIH. Additionally, in contrast to broad‐spectrum anti‐inflammatory drugs, MCC950 does not compromise global immune function due this selective activity. In this study, MCC950 is found to not impact the viability, integrity, or function of human coronary endothelial cells, in contrast to the non‐specific anti‐proliferative effects of PTX and SMS. Using an in vitro model of NLRP3‐mediated inflammation in murine macrophages, MCC950 reduced IL‐1*β* expression, which is a key driver of NIH. In an in vivo mouse model of NIH in vascular grafts, MCC950 significantly enhanced re‐endothelialization and reduced NIH compared to PTX or SMS. These findings show the effectiveness of a targeted anti‐inflammatory drug‐elution strategy with significant implications for cardiovascular device intervention.

## Graphical abstract

Current drug eluting vascular devices releasing paclitaxel and sirolimus prevent growth of key vascular cells but fail to promote vessel healing (endothelialization), resulting in poor long‐term reduction of vessel narrowing (neointimal hyperplasia). In contrast, highly selective anti‐inflammatory MCC950 promotes rapid healing of the blood vessel and reduces vessel narrowing.

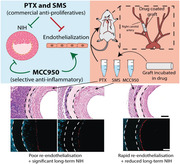

## 1 Introduction

Minimally‐invasive endovascular intervention has become the preferred treatment for certain occlusive cardiovascular diseases, due to low incidences of in‐hospital mortality and faster patient recovery times compared to open surgery.^[^ ^1^ ^]^ These therapies rely on the deployment of balloons and/or stents within diseased blood vessels which push aside plaque deposits and restore blood flow. Despite their acute benefits, endovascular therapies commonly fail in the long‐term due to post‐operative vessel re‐narrowing caused by smooth muscle cell (SMC) invasion of the vessel lumen, a pathological process known as neointimal hyperplasia (NIH).^[^ ^2^ ^]^ Both balloon expansion and stent placement cause collateral damage to the endothelium and vessel wall, triggering a chronic inflammatory injury response.^[^ ^2^ ^]^ Macrophages are the dominant responders to this injury, up‐regulating the secretion of pro‐inflammatory cytokines, namely interleukin‐1*β* (IL‐1*β*), which initiate the phenotypic switching of SMCs in the vessel wall toward a hyperproliferative “synthetic” phenotype.^[^ ^3^ ^]^ Without an intact endothelial barrier, highly proliferative SMCs grow into the vessel lumen, driving NIH and vessel re‐narrowing.^[^ ^4^ ^]^ This has inspired the widespread use of drug‐eluting technology which attempts to minimize NIH by inhibiting SMC growth and extend the acute beneficial outcomes of endovascular intervention.

The evolution of commercial drug‐eluting stents (DES) and drug‐eluting balloons has seen the development of device coatings with water‐insoluble antiproliferative agents including paclitaxel (PTX), sirolimus (SMS), and more recently, SMS family derivatives.^[^ ^5^ ^]^ PTX prevents cell proliferation by promoting the assembly of stable microtubules and inhibiting their depolymerization, leading to cell‐cycle arrest and apoptosis.^[^ ^6^ ^]^ Conversely, SMS inhibits the activation of mammalian target of rapamycin (mTOR), an essential kinase that regulates cell proliferation, arresting cells in the G1 phase of the cell cycle causing a cytostatic effect.^[^ ^7^ ^]^ Although numerous clinical trials have now validated the efficiency of drug‐elution technology in preventing NIH, it is also widely recognized that these drugs compromise the proliferation of many different cell types.^[^ ^8^ ^]^ Drug‐eluting devices fail to address the underlying causes of inflammation, inhibit endothelial cell recovery and healing, and have only limited elution times, ultimately resulting in NIH in the long‐term.^[^ ^9^ ^]^ Furthermore, safety concerns have emerged regarding the use of PTX in particular for drug‐eluting devices, with studies showing an overall increased risk of all‐cause mortality.^[^ ^10^ ^]^ Despite the need for further studies to confirm these findings and determine the mechanisms underlying the increased risk, the non‐specific, cytotoxic nature of these drugs is an important concern relating to safety and efficacy of drug‐eluting approaches. PTX and SMS are also inherently limited as they target only end‐stage vessel re‐narrowing, rather than the early stages of inflammation triggered by the initial injury to the vessel wall.^[^ ^2^ ^]^ The ideal drug would instead simultaneously inhibit SMC proliferation while allowing endothelial cell re‐growth. Recent studies have indicated that drugs which target this initial inflammatory response hold significant promise for improving the long‐term performance of vascular devices.

Despite the well characterized role inflammation plays in NIH and the recent therapeutic links identified between inflammation‐targeting drugs and better cardiovascular outcomes, their use in drug‐eluting devices has yet to be clinically established. An emerging target in vascular inflammation is the NLRP3 (NOD‐, LRR‐, and PYD‐containing protein 3) inflammasome, a cytosolic signaling pathway of the innate immune system responsible for the proteolytic activation of IL‐1*β*.^[^ ^11^ ^]^ Driven by a growing number of studies implicating its role in the pathogenesis of cardiovascular disease and injury, antagonism of the NLRP3 inflammasome is an increasing focus in vascular medicine.^[^ ^11b^ ^]^ MCC950 was the first developed small molecule inhibitor which covalently binds to and prevents NLRP3 oligomerization. In mouse models of atherosclerosis, intravenous MCC950 attenuates IL‐1*β*, significantly reducing atherosclerotic plaque development.^[^ ^12^ ^]^ Further studies in mouse models of myocardial infarction have shown that intraperitoneal injections of MCC950 reduces fibrosis and improves cardiac remodeling.^[^ ^13^ ^]^ More importantly, in vascular healing studies, MCC950 causes no significant impairments to native angiogenesis, suggesting that the selective functions of MCC950 carry robust and targeted anti‐inflammatory actions without the anti‐angiogenic effects of antiproliferative drugs.^[^ ^14^ ^]^ However, despite overlapping inflammatory mechanisms with atherosclerosis, evidence demonstrating the potential of MCC950 and NLRP3 inhibition in suppressing NIH has not yet been reported in the context of vascular devices/materials.

**Figure 1:**
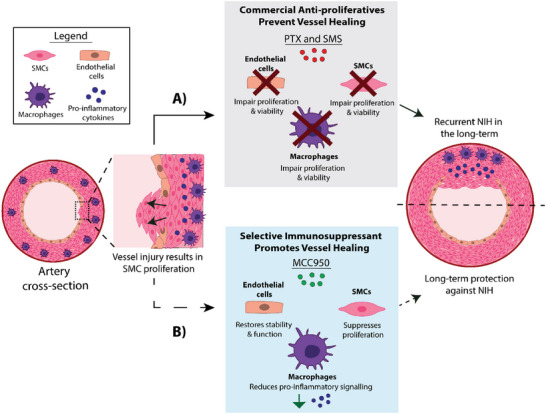
Schematic representation of A) current drug‐eluting endovascular interventions using anti‐proliferative drugs, paclitaxel (PTX) and sirolimus (SMS). Endovascular interventions cause injury to the vessel wall (black arrows), stimulating macrophage‐driven immune responses that causes neointimal hyperplasia (NIH), an over‐proliferation of smooth muscle cells (SMC) into the vessel lumen. Elution of non‐specific anti‐proliferative drugs PTX and SMS reduces NIH in the short‐term, but also negatively impacts endothelial cells and macrophages, preventing vessel healing and leading to long‐term outcomes of recurrent NIH. B) Targeting NLRP3‐mediated inflammation, a potential underlying cause of NIH, using the selective inhibitor MCC950 may suppress NIH without adverse impacts on vessel healing which could ultimately lead to superior long‐term suppression of NIH compared to PTX and SMS.

In this paper, we conduct a comparative study of MCC950 against the established agents PTX and SMS, as a potential alternative for long‐term suppression of NIH. Functioning through an entirely distinct mechanism to PTX and SMS, we first show in vitro that MCC950 is non‐toxic to vascular cells critical to vessel remodeling. MCC950 also selectively reduces expression of inflammatory factors from cultured macrophages which drive NIH while supporting endothelial integrity and function. Further evaluation of MCC950 in a 28 days in vivo vascular graft model of NIH demonstrated superior performance to PTX and SMS across a range of key metrics. MCC950 showed long‐term reduction in vascular inflammation coupled with an early enhancement of endothelial coverage and function. These events were ultimately consistent with a significant and long‐term reduction of NIH and reduced fibrin deposition. These findings collectively highlight MCC950 as a potentially more targeted, effective, and safer drug‐eluting approach for vascular devices (**Figure** **1**A).

## 2 Results

### 2.1 MCC950 Is Non‐Toxic to Vascular Cells

**Figure 2:**
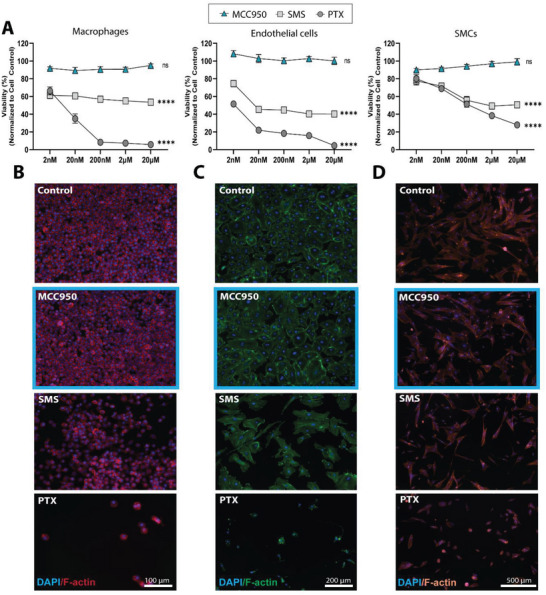
MCC950 is non‐toxic to key vascular cells. A) Viability of J774a murine macrophages, human coronary artery endothelial cells, and smooth muscle cells (SMCs) 3 days after being treated with MCC950, sirolimus (SMS), or paclitaxel (PTX). Data represents mean ± SEM (*n* = 4). Statistical significance was determined using Dunnett's multiple comparison test (^****^ *p* < 0.0001). Representative images of B) macrophages, C) endothelial cells, and D) smooth muscle cells treated with highest dose (20 µm) MCC950, SMS, or PTX. DAPI stained in blue, F‐actin stained in red, green, and light orange, respectively. Scale bars represent 100, 200, and 500 µm, respectively.

The distinct modes of action for each drug were investigated by performing cytotoxicity assays in the three major cell types relevant to the pathophysiology of vessel injury and repair (macrophages, endothelial cells, and SMCs). A viability dosage response curve was generated for each cell type after 3 days of treatment with each drug. Across all cell types both PTX and SMS led to dose‐dependent reductions in viability compared to control (**Figure** **2**A). PTX showed the most significant decreases when dose matched against SMS. Immunostaining showed that when compared to control, PTX and SMS drastically reduced cell density in macrophages (Figure 2B), compromised junction formation in endothelial monolayers (Figure 2C), and impaired cytoskeleton spindles SMCs (Figure 2D). In contrast, MCC950 treated cells had no observable differences compared to control, highlighting that cytotoxicity was not its primary mechanism of action or an adverse side effect. Similar effects were observed in human THP‐1–derived macrophages (Figure S1, Supporting Information).

### 2.2 MCC950 Immunosuppression Is Selective to the NLRP3 Inflammasome In Vitro

**Figure 3:**
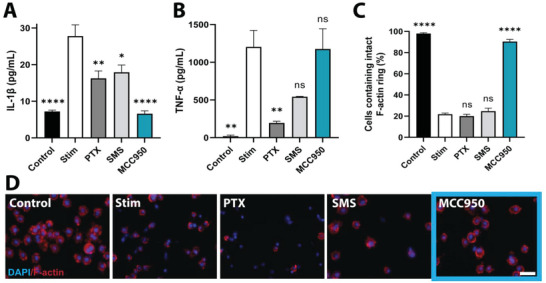
MCC950 selectively suppresses components of the NLRP3 inflammasome pathway in murine macrophages. The effect of high dose (20 µm) MCC950, paclitaxel (PTX), and sirolimus (SMS) on A) IL‐1*β*, B) TNF‐*α* and C) pyroptosis levels post‐NLRP3 inflammasome stimulation with LPS (1 µg mL^−1^) and ATP (2.5 mm) in J774a macrophages. Stim refers to stimulated only group. IL‐1*β* and TNF‐*α* levels measured by ELISA. Data represents mean ± SEM (*n* = 3–4). Statistical significance was determined using Dunnett's multiple comparison test relative to stimulated group (^*^ *p* < 0.05, ^**^ *p* < 0.01, ^***^ *p* < 0.001, ^****^ *p* < 0.0001). D) Representative images of control, stimulated only, and stimulated and drug treated macrophages used for pyroptosis quantification. Cells stained with DAPI (blue) and rhodamine phalloidin (red) to visualize cell nucleus and F‐actin, respectively. Scale bar represents 50 µm.

Validation of MCC950 function was conducted by establishing an in vitro model of NLPR3‐mediated inflammation. Stimulated macrophages were treated with each drug for 24 h followed by quantification of IL‐1*β* secretion and pyroptosis‐mediated cell death, the major products of NLRP3 inflammasome activation (Figure S2, Supporting Information). TNF‐*α* secretion was also quantified to serve as a control inflammatory cytokine non‐specific to the NLRP3 pathway (TNF‐*α* is collaterally secreted by the priming LPS stimulus as the first component of NLRP3 activation). PTX and SMS treatment showed broad suppression of inflammatory cytokine release, reducing both IL‐1*β* and TNF‐*α* (**Figure** **3**A,B). However, neither drug was able to elevate levels of intracellular F‐actin staining relative to stimulated groups, indicating an inability to rescue macrophages from pyroptosis (Figure 3C). In contrast, MCC950 showed markedly reduced levels of IL‐1*β* relative to stimulated control and to a greater degree than both PTX and SMS (6.6 ± 0.76 vs 27.78 ± 3.08, 16.27 ± 2.02, and 17.91 ± 2.0 pg mL^−1^, respectively). Additionally, MCC950 alone rescued stimulated macrophages from pyroptosis raising F‐actin levels by 76% above stimulated controls (90.5 ± 1.98% vs 21.77 ± 1.13%). MCC950 had no effect on TNF‐*α* secretion, highlighting that its anti‐inflammatory functions were specific to the NLRP3 pathway. Similar observations, though with a higher degree of variability, were recorded in human THP‐1–derived macrophages due to the non‐adherent nature of this cell type, where MCC950 inhibited IL‐1*β* secretion and rescued pyroptosis, with negligible effect on TNF‐*α* (Figure S3, Supporting Information).

### 2.3 MCC950 Does Not Compromise Endothelial Cell Integrity or Function In Vitro

**Figure 4:**
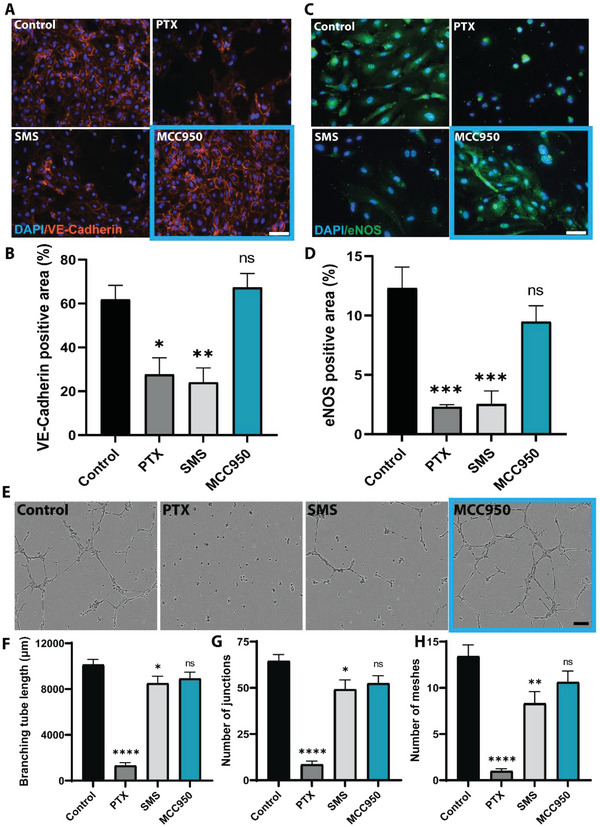
MCC950 does not impair endothelial functionality in vitro. A) Representative images of human coronary artery endothelial cells 3 days after treatment with high dose (20 µm) paclitaxel (PTX), sirolimus (SMS), or MCC950. DAPI stained in blue, vascular endothelial (VE)‐Cadherin in orange. Scale bar represents 100 µm. B) Quantification of total VE‐cadherin staining represented as a percentage of total area. C) Representative images of human coronary artery endothelial cells 3 days after treatment with paclitaxel (PTX), sirolimus (SMS), or MCC950. DAPI stained in blue, endothelial nitric oxide synthase (eNOS) in green. Scale bar represents 50 µm. D) Quantification of total eNOS staining represented as a percentage of total area. E) Representative images of HCAEC tubule formation assay following 6 h treatment with high concentration (20 µm) paclitaxel (PTX), sirolimus (SMS), or MCC950. Scale bar represents 150 µm. F–H) Quantitative analysis of tubule formation assay presented as G) branching tube length, H) number of tubule junctions formed, and I) number of tubule meshes formed. Data represents mean ± SEM (*n* = 3–4). Statistical significance was determined using Dunnett's multiple comparison test against control group (^*^ *p* < 0.05, ^**^ *p* < 0.01, ^***^ *p* < 0.001, ^****^ *p* < 0.0001).

The major limitation of anti‐proliferative drugs like PTX and SMS are their non‐specific cytotoxic effects which compromise functional re‐endothelialization and subsequent vessel healing. To determine any negative effects MCC950 may have directly on endothelial cells, levels of vascular endothelial‐cadherin (VE‐cadherin) and endothelial nitric oxide synthase (eNOS) were measured to assess endothelial integrity and regulatory function. Compared to untreated controls, VE‐cadherin expression was reduced at junctions between neighboring endothelial cells after treatment with PTX or SMS by 55% and 61%, respectively (**Figure** **4**A,B), an effect which appeared to be dose‐dependent (Figure S4A, Supporting Information). MCC950 however showed strong VE‐cadherin staining with no quantifiable differences in expression compared to control. Similarly, cells treated with PTX or SMS showed an 81% and 79% reduction in eNOS expression, respectively, in contrast to MCC950 which maintained levels of eNOS comparable to control groups (Figure 4C,D). The effects of PTX and SMS were also dose dependent (Figure S4B, Supporting Information). Additional analysis using real‐time qPCR of typical endothelial phenotype and function genes cadherin 5 (CDH5), platelet endothelial cell adhesion molecule (PECAM), and eNOS further supported the compatibility of MCC950 by showing no downregulation of these genes (Figure S4C–E, Supporting Information). Collectively, this suggested that MCC950 had negligible adverse effects on endothelial cells.

To further investigate the effects of MCC950 on endothelial function over PTX and SMS, a HCAEC tube formation assay was performed (Figure 4E). Both PTX and SMS significantly impaired tubule formation, decreasing branching tube length, number of tube junctions, and number of tube meshes formed (Figure 4F). This contrasted with MCC950 which showed no significant reduction in any of these outcomes. This further highlighted that MCC950 does not compromise endothelial cell integrity or function, suggesting that it may not have the negative impacts on re‐endothelialization in vivo known for PTX and SMS.

### 2.4 MCC950 Improves Hemocompatibility In Vitro

**Figure 5:**
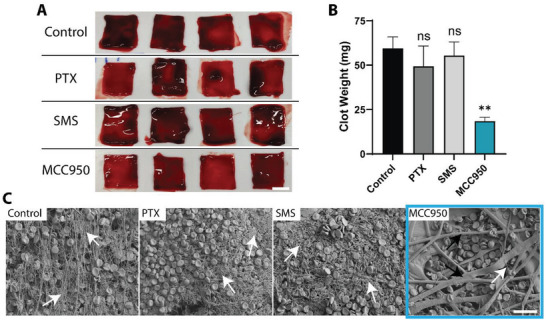
MCC950 shows greater hemocompatibility compared to commercial agents PTX and SMS. A) Representative images of human whole blood clotting assay using high porosity silk scaffolds treated with 30 µm paclitaxel (PTX), sirolimus (SMS), or MCC950 for 1 h. Scale bar = 0.5 cm. B) Quantitative analysis of blood clot weight following 1 h drug incubation. C) Representative scanning electron micrograph images of silk scaffolds following clotting assay. White arrows demonstrate fibrin networks and black arrows show the underlying silk scaffold fibers. Scale bar = 20 µm. Data represents mean ± SEM (*n* = 2–4). Statistical significance was determined using Dunnett's multiple comparison test against control group (^**^ *p* < 0.01, ^***^ *p* < 0.001).

Drug hemocompatibility, a key feature of eluted agents used in vascular settings, was studied using an established in vitro human whole blood clotting assay. Fresh human blood was added to silk scaffolds and treated with PTX, SMS, or MCC950.^[^ ^15^ ^]^ Scaffolds were then washed and their surface analyzed to determine the weight of clotting blood (**Figure** **5**A). No significant differences in clot weight were observed after PTX or SMS treatment compared to control, whereas MCC950 significantly reduced clotting weight by 69% (Figure 5B). SEM imaging of these samples was conducted to qualitatively examine fibrin network formation, a critical precursor event to thrombus formation (Figure 5C). In these experiments, PTX and SMS again were indistinguishable from control whereas MCC950 showed a substantial decrease in fibrin networks. Taken together, these findings demonstrated the improved hemocompatibility of MCC950 over PTX and SMS.

### 2.5 MCC950 Suppresses Vascular Inflammation In Vivo

**Figure 6:**
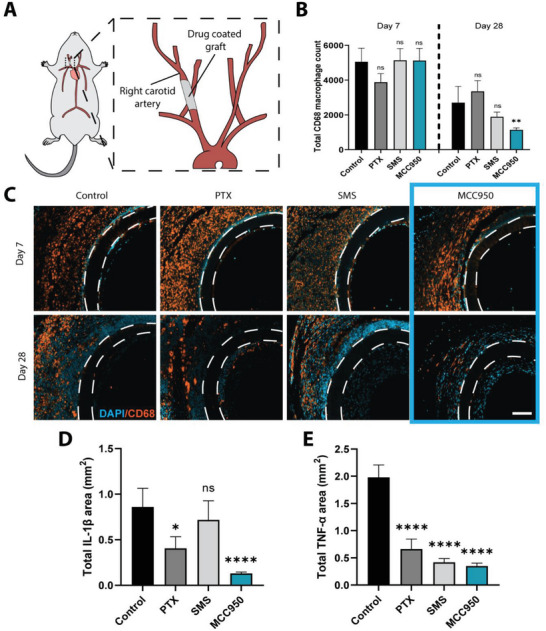
MCC950 strongly suppresses vascular inflammation. A) Schematic representation of the in vivo interposition vascular graft model used in this study. B) Quantification of total number of CD68^+^ cells. C) Representative images of CD68 stain taken from middle region of explanted grafts. DAPI stained in blue, CD68 stained in orange. Scale bar represents 100 µm. D,E) Quantification of total IL‐1*β* (D) and TNF‐*α* (E) positively stained area at day 28. Data represents mean ± SEM (*n* = 3–4). Statistical significance was determined using Dunnett's multiple comparison test (^*^ *p* < 0.05, ^**^ *p* < 0.01, ^****^ *p* < 0.0001).

Drug evaluation was next performed in vivo by passively adsorbing each drug onto vascular grafts prior to implantation in a mouse carotid interposition model of graft healing and NIH for 28 days (**Figure** **6**A). To test the anti‐inflammatory effects of MCC950 in vivo, macrophage recruitment was quantified by CD68^+^ immunostaining to first determine changes to local vascular inflammation (Figure 6B). From day 7 to 28, macrophage recruitment was found to decrease across all groups (Figure 6C). Significant differences occurred at day 28 only in grafts treated with MCC950 which showed an average 57% decrease in total macrophages compared to control. The majority of these macrophages were observed on the exterior of the graft surface. The effects on vascular inflammation were further examined by analyzing IL‐1*β* and TNF‐*α* expression (Figure 6D,E and Figure S5, Supporting Information). By day 28, MCC950 had the largest significant reductions in IL‐1*β* expression followed by PTX when compared to control (0.13 ± 0.01 and 0.41 ± 0.13 vs 0.96 ± 0.2 mm^2^, respectively) (Figure 6D). For TNF‐*α*, all drugs led to significant reductions compared to control (0.66 ± 0.18, 0.42 ± 0.12, and 0.35 ± 0.12 vs 1.98 ± 0.34 mm^2^, respectively) with the most significant reductions occurring in the MCC950 groups (Figure 6E). Collectively, these results suggested MCC950 was more effective at achieving comprehensive suppression of local inflammation.

### 2.6 MCC950 Promotes Re‐Endothelialization In Vivo

**Figure 7:**
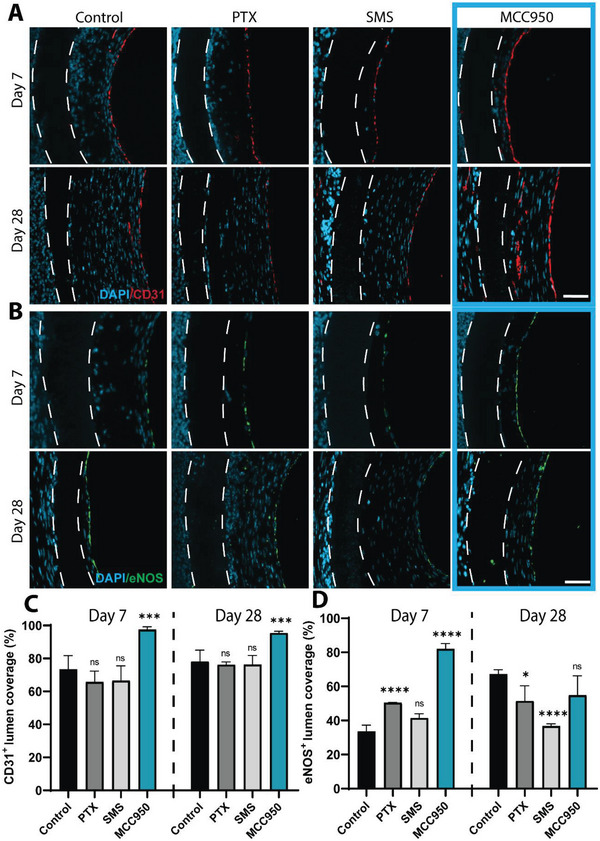
MCC950 promotes rapid re‐endothelialization. A,B) Representative images of CD31^+^ (A) stained sections in red and eNOS^+^ (B) stained sections in green taken from middle portion of explanted grafts after 7 and 28 days. Scale bar represents 50 µm. C,D) Quantification of CD31^+^ (C) and eNOS^+^ (D) staining at day 7 and day 28. Data represents mean ± SEM (*n* = 2–3). Statistical significance was determined using Dunnett's multiple comparison test against control (^***^ *p* < 0.001, ^****^ *p* < 0.0001).

To determine the effects of each drug on functional endothelialization, grafts were immunostained for endothelial coverage (CD31) and function (eNOS), respectively. Endothelialization peaked at day 7 with no changes by day 28 in all grafts (**Figure** **7**A). Control, PTX, and SMS treated grafts showed patchy and incomplete CD31^+^ endothelial coverage of the graft lumen at day 7 when compared to the endothelial layer in MCC950 groups which appeared almost complete. This coverage was quantified at ≈97 ± 1.8% of the total graft lumen, a 25% increase compared to control (Figure 7C). Similarly, at day 7, low levels of eNOS^+^ staining were evident across the luminal surface of control, PTX and SMS grafts compared to grafts with MCC950 which showed almost complete positive staining (Figure 7B). Over 28 days eNOS was found to increase in control grafts. Notable changes in eNOS^+^ coverage was observed at day 7, where both PTX and MCC950 showed increased recovery of eNOS function compared to control, although to a significantly higher degree in MCC950 groups (50.49 ± 0.04% and 82 ± 3.14% vs 33.62 ± 3.65%, respectively) (Figure 7D). By day 28 these increases had largely resolved with both PTX and SMS showing decreased eNOS^+^ coverage compared to control. In contrast, eNOS^+^ coverage in MCC950 had returned to control levels. These findings suggest that MCC950 promotes more rapid and functional endothelialization.

### 2.7 MCC950 Suppresses Fibrotic Encapsulation and Neointimal Hyperplasia In Vivo

**Figure 8:**
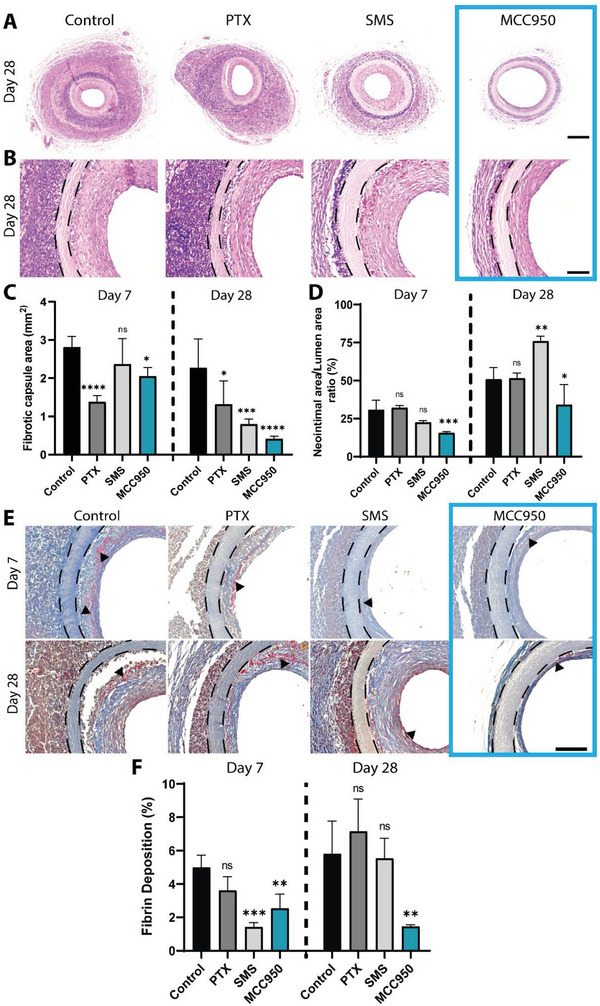
MCC950 reduces fibrotic encapsulation, neointimal hyperplasia, and fibrin deposition. A) Representative images of hematoxylin and eosin‐stained grafts at day 28 showing fibrotic capsule surrounding the graft. Scale bar represents 300 µm. B) Representative images of hematoxylin and eosin‐stained grafts at day 28 showing neointimal hyperplasia. Black dotted lines show graft outline. Scale bar represents 100 µm. C) Quantification of fibrotic capsule area surrounding graft. D) Quantification of neointimal area expressed as a percentage of total lumen area. E) Representative brightfield images of Martius Scarlet Blue stain. Mature fibrin is stained in red (demonstrated with black arrows), fresh fibrin in yellow, and collagen in blue. Scale bar = 100 µm. F) Quantification of mature fibrin (red) deposition within the neointima at days 7 and 28, represented as a percentage of the positive staining area versus total lumen area. Data represents mean ± SEM (*n* = 2–4). Statistical significance was determined using Dunnett's multiple comparison test against control (^*^ *p* < 0.05, ^**^ *p* < 0.01, ^***^ *p* < 0.001, ^****^ *p* < 0.0001).

The ultimate functional outcomes of each drug were assessed by measuring the development of fibrotic encapsulation and NIH using haematoxylin and eosin (H&E) staining (**Figure** **8**A,B). The area of the fibrotic capsule surrounding control grafts decreased marginally between day 7 and day 28 (Figure S6A, Supporting Information). Compared to controls, PTX showed a 51% reduction in capsule area at day 7 which was largely maintained by day 28 (1.38 ± 0.16 vs 2.8 ± 0.28 mm^2^, Figure 8C). SMS had no effect on capsule area at day 7 but was found to decrease capsule area by 65% by day 28 (0.8 ± 0.14 vs 2.3 ± 0.76 mm^2^). Similar to PTX groups, MMC950 showed increasing reductions across both timepoints. However, despite a smaller a 27% reduction at day 7, MCC950 showed a much larger reduction of 81% at day 28.

Neointima occlusion in the graft lumen of control groups was also found to increase over 28 days (Figure S6B, Supporting Information). PTX treatment showed no significant difference to control groups at any timepoint while SMS showed a 33% increase in neointima occlusion compared to control at day 28 (75.84 ± 3.34% vs 50.88 ± 7.68% occlusion) (Figure 8D). Interestingly, at both days 7 and 28, treatment with MCC950 significantly reduced neointimal occlusion by 49% and 33%, respectively, compared to control (15.64 ± 0.9% vs 30.75 ± 6.29% and 34.13 ± 13.23% vs 50.88 ± 7.68% occlusion). Collectively, this suggested that by suppressing the inflammatory microenvironment surrounding the graft, MCC950 was able to achieve better long‐term suppression of NIH.

Fibrin deposition within the graft lumen was also analyzed using Martius Scarlet Blue staining as an additional measure of hemocompatibility and indicator of potential thrombosis (Figure 8E). Over the 28 days, fibrin deposition increased across all groups except for grafts treated with MCC950, which showed a sustained reduction in fibrin (Figure 8F). Compared to control, MCC950 significantly decreased fibrin by 49% and 74% at day 7 and 28, respectively. Although SMS decreased fibrin deposition at day 7, it was unable to sustain this effect at day 28. PTX showed no significant reduction in fibrin levels at either time point compared to control.

## 3 Discussion

Current drug‐eluting endovascular balloons and stents using anti‐proliferative agents PTX and SMS largely fail to address the underlying inflammation which drives NIH. This leads to poor long‐term patency and frequent re‐intervention in areas of aggressive NIH, such as the legs.^[^ ^9^ ^]^ Additionally, in the case of PTX use in peripheral applications, high doses and long elution times have led to increases in amputation rates and mortality, highlighting the need for a safer, more effective drug‐eluting strategy with more durable benefits.^[^ ^10^ ^]^ The NLRP3 inflammasome is a component of the innate immune system with growing evidence for its involvement in vascular inflammation specific to athero‐occlusive cardiovascular diseases.^[^ ^16^ ^]^ However, examination of the NLRP3‐inflammasome as a potential drug‐eluting target for endovascular devices and vascular injury has not yet been studied. Here, we evaluate antagonism of the NLRP3 inflammasome using the selective small molecule inhibitor MCC950, in comparison to commercial agents used in drug‐eluting devices, PTX and SMS.

Driven by non‐specific anti‐proliferative functions acting broadly on all cells present within the vessel wall and lumen, PTX and SMS compromise long‐term vessel inflammation and healing.^[^ ^17^ ^]^ Consistent with these actions, these drugs caused a dose‐dependent decrease in cell viability within macrophages, endothelial cells, and SMCs in vitro. In agreement with clinical observations, this effect was most notable in PTX. PTX action as a cytotoxic agent is strategically utilized to treat more aggressive cases of NIH occurring in settings such as peripheral arterial disease.^[^ ^18^ ^]^ Additionally, PTX usage comes with a trade‐off between effective suppression of NIH and patient safety. Its significant cytotoxic effects can be detrimental to organs downstream from the vasculature from where its delivered.^[^ ^10^ ^]^ SMS showed similar reductions in cell viability but to a lesser extent. This was most likely due to its cytostatic rather than cytotoxic mechanism of action, which halt cell division rather than promoting cell death.^[^ ^7^ ^]^ These features make SMS beneficial in regions such as the coronary arteries where NIH is less aggressive and allows clinicians to prioritize patient safety.^[^ ^8b^ ^]^ However, these collective anti‐proliferative effects are of greatest impact to endothelial cells, highlighting safety limitations in preventing short‐term vessel healing and increased risk of thrombosis, demonstrated by the poor clinical performance of first‐generation DES in coronary applications.^[^ ^19^ ^]^ In contrast, MCC950 showed negligible cytotoxic effects on all tested cell lines. This is the first direct evidence to show biosafety of MCC950 on vascular endothelial cells, macrophages, and SMCs in direct comparison to PTX and SMS. Support of MCC950 cytocompatibility has increasingly been reported, previously shown to be non‐toxic to human kidney HEK293 and liver HepG2 cell lines.^[^ ^20^ ^]^ More broadly, these results also suggest that MCC950 functions through mechanisms distinct from blocking proliferation, representing a fundamental departure from PTX and SMS.

Further examination of MCC950 mechanism of action was performed in macrophages using an immune activation model that aims to model the NLRP3‐inflammasome. LPS stimulation was used as a priming signal to activate TLR4 receptors, triggering the secretion of TNF‐*α* and initiating the transcription of the NLRP3 protein. Secondary co‐stimulation with ATP facilitated the oligomerization/activation of the NLRP3 inflammasome leading to the secretion of IL‐1*β* and initiation of pyroptotic cell death. Quantification of TNF‐*α* was used as a surrogate measure of systemic inflammation while IL‐1*β* and rate of pyroptosis was measured as a specific output of NLRP3‐mediated inflammation. In this model, both PTX and SMS decreased IL‐1*β* and TNF‐*α*, but showed no effect on pyroptosis. These results can be largely attributed to the broad effects these two drugs are known to have on inflammation. In an LPS‐induced liver injury model in mice, PTX was shown to decrease levels of TNF‐*α*, IL‐1*β* and IL‐6 by upregulating miR‐27a, a known inhibitor of cell proliferation and systemic inflammation.^[^ ^21^ ^]^ Similarly, SMS has been reported to down‐regulate widely acting inflammatory cytokines including IL‐6 and TNF‐*α* in THP‐1 macrophages treated with LPS.^[^ ^22^ ^]^ Although the immunomodulatory effects of SMS are still poorly understood, some reports have indicated that the drug can increase sirtuin 1 which leads to decreased NF‐*κ*B activity, reducing pro‐inflammatory cytokine production.^[^ ^22^, ^23^ ^]^ In contrast, MCC950 showed no effect on TNF‐*α* secretion but instead reduced IL‐1*β* secretion and prevented pyroptosis only. As exclusive products of NLRP3‐inflammasome activation, this validated that MCC950 was highly selective for NLRP3‐mediated inflammation. Previous studies have similarly validated inhibition of the NLRP3 inflammasome without affecting other elements of the innate immune system including activation of other inflammasomes such as AIM2, NLRC4, or NLRP1.^[^ ^24^ ^]^ Our study evaluated both murine and human derived macrophages, and found the trends to be similar. However, consistent with previous studies, we observed that the murine derived cell line, being adherent and more responsive to stimuli, yielded less variability.^[^ ^25^ ^]^ Collectively, our results demonstrate the targeted anti‐inflammatory functions of MCC950 on NLRP3‐mediated inflammation and further highlight its distinction from PTX and SMS.

Further evidence of the potential benefits of MCC950 as an alternative drug‐eluting approach was observed in endothelial integrity and function assays. In response to vascular injury, locally derived endothelial cells and endothelial progenitor cells repair and repopulate the endothelium.^[^ ^26^ ^]^ However, delayed re‐endothelialization is common in the context of drug‐eluting devices. In endothelial cells in vitro, PTX and SMS caused reductions in the expression of vascular endothelial cadherin (VE‐cadherin) and eNOS, indicating impairment to critical aspects of endothelial recovery. VE‐cadherin is an endothelial specific adhesion molecule essential to controlling the integrity and permeability of junctions within the healing vessel endothelium.^[^ ^26^ ^]^ The healing endothelium also requires functional eNOS to synthesize nitric oxide which plays numerous central roles from the regulation of vascular tone to injury recovery.^[^ ^27^ ^]^ Reduced expression of both markers are key indicators of endothelial dysfunction. MCC950 alone preserved the expression of both VE‐cadherin and eNOS, suggesting its improved suitability for endothelial recovery. This was supported with transcriptional analysis of CDH5, PECAM, and eNOS genes that showed MCC950 caused no significant reductions in their expression compared to control. Further analysis using the established Matrigel‐based tubule formation assay^[^ ^28^ ^]^ to examine endothelial function exemplified the negative effects of both PTX and SMS on endothelial recovery. Both drugs function mechanistically to disrupt cell growth and division, preventing neo‐capillary formation. In contrast, MCC950 had no impact on tubule formation, demonstrating a clear distinction between its mechanism of action which had no adverse effects on endothelial cells and showcases its potential for improved re‐endothelialization in vivo.

Hemocompatibility and the risk of thrombosis is a major concern for all vascular devices. Current devices that employ PTX or SMS can lead to high rates of late‐stage thrombosis due to poor re‐endothelialization and excessive fibrin accumulation in the vessel wall.^[^ ^29^ ^]^ Drugs eluted from these devices should ideally have high hemocompatibility and can carry additional benefit by reducing the risk of thrombosis. In human whole blood clotting assays, MCC950 decreased clotting weight and fibrin accumulation. Previous studies have shown the NLRP3 inflammasome to be activated in platelets during thrombosis resulting in the secretion of both IL‐1*β* and tissue factor (TF).^[^ ^30^ ^]^ IL‐1*β* along with other mediators are known to promote platelet activation and aggregation, while TF helps to initiate the coagulation cascade by increasing platelet production of fibrinogen, the precursor to fibrin.^[^ ^31^ ^]^ Our study is in agreement with previous research suggesting a link between NLRP3 inhibition and reduced arterial thrombosis. However, our findings are the first to directly compare the efficacy of MCC950 with commercial drug standards PTX and SMS.

Extending these findings to the in vivo, drug evaluation was conducted using vascular grafts passively absorbed with each drug prior to implantation in an established mouse carotid interposition grafting model of NIH for 7 and 28 days.^[^ ^32^ ^]^ Vascular inflammation was first assessed by quantifying macrophages. Macrophages resolved over the 28 days, however PTX and SMS showed no differences in macrophage levels compared to control. In contrast, MCC950 enhanced resolution of macrophages by day 28, indicative of strong anti‐inflammatory effects. As the principal drivers of vascular inflammation, large populations of macrophages surrounding vascular lesions are associated with increased risk of NIH.^[^ ^33^ ^]^ Surprisingly, this was not entirely associated with pro‐inflammatory cytokine expression. Both PTX and SMS showed reductions in IL‐1*β* and TNF‐*α* despite no significant changes to macrophage content. This could potentially be explained by increased macrophage retention by PTX and SMS, compared to MCC950 which enhanced macrophage resolution. Previous studies have suggested that both PTX and SMS shift the balance of macrophage polarization toward the pro‐inflammatory M1 phenotype directly via the TLR4 and mTOR pathways, respectively, independent of cytokine stimuli and/or cytokine production.^[^ ^34^ ^]^ M1 macrophages are enriched within inflamed tissue until signaled to resolve by anti‐inflammatory cytokines. Increased macrophage retention together with enhanced cellular dysfunction because of PTX and SMS treatment could also explain how cytokine levels were lowered. Compared to PTX and SMS, MCC950 showed even greater reductions of both IL‐1*β* and TNF‐*α* together with reduced macrophage levels. As the major product of NLRP3‐mediated inflammation, IL‐1*β* promotes the recruitment and retention of macrophages during inflammatory conditions.^[^ ^35^ ^]^ In agreement with our observations, a previous study showed MCC950 decreased infiltrating CD68^+^ macrophages in a mouse model of myocardial infarction and suggested IL‐1*β* suppression was, at least in part, a driving factor.^[^ ^13^ ^]^ However, contrary to previous in vitro findings, MCC950 also reduced TNF‐*α*. This was suggestive of indirect anti‐inflammatory effects that reflect a broader immunosuppressive effect. These outcomes demonstrate the widespread effects MCC950 had on the local inflammatory microenvironment surrounding the graft.

Re‐endothelialization is a critical step in healing and reducing NIH in the long term.^[^ ^36^ ^]^ Despite the short‐term benefits PTX and SMS have on NIH, a limitation of these anti‐proliferative agents is delayed re‐endothelialization.^[^ ^37^ ^]^ We chose the mouse grafting model to study this as it has been previously well characterized and demonstrated to be a highly reproducible model of re‐endothelialization in a compressed 28‐day time frame, making it ideal for comparative studies identifying promising new candidate molecules.^[^ ^32^ ^]^ Here, endothelial coverage was found to occur prior to the re‐establishment of endothelial function. PTX and SMS showed no differences from control at day 7 or 28, indicating that both drugs had only achieved baseline levels of re‐endothelialization. This was also associated with reduced eNOS function in both groups by day 28, suggesting that both drugs were impairing the re‐establishment of endothelial function. Contrary to these findings, MCC950 grafts not only had the highest levels of endothelial coverage, achieving near complete re‐endothelialization by day 7, but also enhanced rates of early eNOS expression compared to control. Antagonism of NLRP3 has been previously linked to similar endothelial protective effects. NLRP3 inflammasome‐mediated pyroptosis has been shown to closely associate with endothelial membrane rupture and cell lysis, releasing various cellular contents including pro‐inflammatory cytokines and high‐mobility group box 1 which further exacerbate endothelial dysfunction by increasing cell permeability and disrupting endothelial junctions.^[^ ^38^ ^]^ These effects are likely to occur naturally in control implants potentially explaining the enhanced re‐endothelialization of MCC950 grafts compared to control. These results are the first to show positive re‐endothelialization effects due to MCC950 in the context of materials implanted directly into the vasculature. Interestingly, these enhanced rates of eNOS expression had resolved back down to control levels by day 28. This may suggest that these effects are dependent upon MCC950 acting directly upon endothelial cells, rather than indirectly through changes to macrophages and the local immune microenvironment.

Inflammatory processes drive fibrotic capsule formation and NIH, the main biological causes of endovascular device failure.^[^ ^39^ ^]^ Minimizing fibrotic encapsulation is associated with a suppressed local inflammatory response and has been closely linked to end‐stage tissue healing.^[^ ^40^ ^]^ Following stent implantation, a fibrous capsule made up of mostly collagen fibers forms around the implant and induces the progression of NIH.^[^ ^41^ ^]^ Consistent with the benefits MCC950 showed in suppressing inflammatory conditions discussed above, we also demonstrated the striking reductions MCC950 had in fibrotic capsule development, further highlighting its ability to reduce the inflammatory microenvironment and promote healthy vascular remodeling. In addition, contrary to clinical observations, PTX and SMS showed no reductions in NIH in our model. Given that this carotid grafting model is an accelerated model of NIH, the dosage and rate of drug delivery may have been insufficient for both drugs, while their usual physiological mode of action is heavily dependent on absorption into the native vascular wall. In our context, SMS appeared to worsen hyperplasia development at day 28. However, the aggressive formation of NIH in this model did demonstrate the striking impact of NLRP3‐antagonism and MCC950. In this model, MCC950 was comparatively stronger at suppressing hyperplasia, showing significant reductions as early as day 7 which persisted to day 28. Hemocompatibility is a critical factor that affects the long‐term performance of vascular materials. When fibrin accumulates on the material surface, it increases the risk of thrombus formation which can obstruct blood flow and potentially lead to life‐threatening conditions. PTX and SMS were unable to sustain a reduction in fibrin deposition within the neointima, while MCC950 significantly decreased fibrin levels at day 28. Fibrin deposition and re‐endothelialization are closely related, as excessive deposition can impede the re‐endothelialization process. Rapid endothelialization on the other hand is associated with a reduced risk of fibrin deposition. Achieving rapid re‐endothelialization is a key strategy to improving the long‐term performance of vascular materials and devices both in limiting NIH development, fibrin deposition, and thrombus formation.^[^ ^42^ ^]^ Coupled with enhanced rates of re‐endothelialization, these results showcase the potential long‐term benefits of MCC950. Collectively, these in vivo findings represent crucial proof‐of‐concept data for the therapeutic benefit of a selective immunosuppressive MCC950 approach. This has important implications for stenting and balloon applications with this study justifying further testing and validation of our approach in established large animal models specific to these applications.

## 4 Conclusion

This study demonstrated the advantages of a selective anti‐inflammatory approach using the NLRP3‐inflammasome inhibitor MCC950 over the current clinical standards for reducing NIH employing anti‐proliferative drugs. Compared to the non‐specific function of PTX and SMS, MCC950 does not impair the viability or function of endothelial cells, allowing re‐endothelialization and vessel healing to occur. By instead selectively targeting NLRP3‐mediated inflammation, MCC950 exhibited a robust suppression of the inflammatory microenvironment surrounding implanted vascular grafts leading to sustained reductions of NIH. Our findings are the first to demonstrate the effectiveness of MCC950 as a drug‐elution strategy for materials implanted in the vasculature. Further validation of this targeted anti‐inflammatory approach in large animal models is now warranted.

## 5 Experimental Section

### Cell Culture

Murine macrophages (J774a.1, passage 3–6, Sigma Aldrich, MA, USA), SMCs (CSC‐C4357X, passage 4–6, Creative Bioarray, Shirley, NY, USA), human coronary artery endothelial cells (HCAECs, 300–05a, passage 4–6, Cell Applications, San Diego, CA, USA), and THP‐1 (passage 3–6, Sigma Aldrich, MA, USA) were maintained at 37 °C in a 5% CO_2_ humidified incubator. Macrophages were cultured in Dulbecco's Modified Eagle Medium (10 mL,) supplemented with Fetal Bovine Serum (10% v/v,) and 1% antibiotics (100 U mL^−1^ Penicillin and 100 µg mL^−1^ Streptomycin), SMCs in SMC medium (Merck, Darmstadt, Germany), HCAECs in Mesoendo medium (Merck, Kenilworth, NJ, USA), and THP‐1s in RPMI‐1640 medium (Merck, Kenilworth, NJ, USA). The cell culture media was replaced with fresh media every 2 – 3 days. Cells were subcultured after reaching ≥85% confluency by first aspirating the old medium, washing with Phosphate‐Buffered Saline (10 mL, PBS), and adding fresh medium (10 mL). A cell scraper was used to mechanically detach macrophages from the flask while SMCs and HCEACs were trypsinized. THP‐1s were aspirated from flask. Resulting cell suspension was transferred to a falcon tube for centrifugation (1200 rpm, 5 min). The supernatant was aspirated, and the pellet was resuspended in fresh medium (2–5 mL, depending on size). Cell concentration was determined by staining with Trypan Blue (1:1) and manually counting cells using a haemocytometer. The cell suspension was triturated before seeding the required volume onto a new T75 flask and adding fresh medium (10 mL).

### Cell Viability Assay

Cells were seeded into 96‐well plates at a density of 3 × 10^4^ cells per well followed by immediate treatment with drug groups (200 µL medium in total with drug) at specified concentrations. For THP‐1s, cells were treated with phorbol 12‐myristate 13‐acetate (PMA, 100 ng mL^−1^) for 24 h before seeding. Cultures were left to incubate for 3 days. Cell viability was quantified using an Alamar Blue assay at a ratio of 1:10 with fresh cell culture media. Plates were incubated for 3 h and quantified using a microplate reader (Infinite M1000 PRO) for fluorescence analysis. Excitation and emission wavelengths were set at 560 and 590 nm, respectively. Complementary morphological analysis of treated cultures was conducted at 3 days post‐seeding. Cells were rinsed twice with PBS prior to fixation with 10% formalin for 10 min and an additional two 5 min washes in PBS. Cells were then permeabilized using a Triton X‐100 detergent (0.1% in PBS) for 10 min followed by two 5 min washes in PBS. Cell nuclei were fluorescently stained using NucBlue Fixed Cell ReadyProbes Reagent (DAPI, ThermoFisher Scientific, 1 drop per mL PBS) and F‐actin with ActinRed 555 Reagent (Rhodamine phalloidin, ThermoFisher Scientific, 1 drop per mL PBS). Cells were visualized and imaged using Zen inverted fluorescent microscope.

### NLRP3 Inflammasome Activation Assay

J774a.1 murine macrophages were seeded onto a 24‐well plate at a density of 1.2 × 10^5^ cells per well. Following pre‐treatment with PMA (100 ng mL^−1^) for 24 h, THP‐1s were also seeded at the same density as murine macrophages. NLRP3 inflammasome activation was achieved in two steps. First, the inflammasome was primed by stimulating cells with lipopolysaccharide (1 µg mL^−1^) for 3 h at 37 °C, inside the humidified incubator (5% CO_2_). After 90 min, drugs were added at the specified range of doses. After an additional 90 min (3 h post‐LPS stimulation), cells were stimulated with ATP (1.25 mm) to activate the inflammasome and left in the incubator overnight. Enzyme‐linked immunosorbent assay (ELISA) kits for TNF*α* (Abcam, ab208348 and ab181421) and IL‐1ß (Abcam, ab197742 and ab214025) were used to quantify cytokine levels in supernatants according to manufacturer's instructions. Pyroptosis was evaluated through measurement of F‐actin degradation by co‐staining cells with DAPI and Rhodamine Phalloidin, as described above. Percentage of cells undergoing pyroptosis was quantified by manually counting DAPI positive cell nuclei containing an intact F‐actin ring and normalized against total amount of DAPI positive cell nuclei.

### In Vitro Endothelial Integrity Assay

Endothelial cell integrity was assessed using immunohistochemistry. HCAECs were seeded as per cell viability protocols and on day 3 were similarly fixed and permeabilized as described above. Cells were then blocked with bovine serum albumin (BSA, 5% in PBS‐Tween 20) for 1 h at room temperature. After blocking, cells were stained with primary antibodies against vascular endothelial (VE)‐cadherin (1:200, ab33168, Abcam) or endothelial nitric oxide synthase (eNOS) (1:100, ab76198, Abcam) for 48 h at 4 °C. Cells were then washed with PBST (2 × 10 min) and secondary antibody (1:500, ab150077, Abcam) was added for 1 h at room temperature. After a series of washes with PBST (2 × 10 min) and PBS (1 × 5 min), DAPI solution (ThermoFisher Scientific, 1 drop per mL PBS) was added. Cells were left to rest in the dark for 10 min before being visualized and imaged using Zen inverted fluorescent microscope. Using a constant threshold, amount of VE‐cadherin and eNOS positive staining was measured as a percentage of total area.

### qPCR

Quantitative PCR was performed using iQ SYBR‐Green Supermix and the iCycler real‐time PCR detection system (Bio‐Rad). Angiogenesis‐related gene expression was calculated using primers for eNOS (forward, 5′‐CGGAGAATGGAGAGAGCTTTG‐3′; reverse, 3′‐TGCTGTTGAAGCGGATCTTA‐5′), CDH5 (forward, 5′‐CGCAATAGACAAGGACATAAC‐3′; reverse, 3′‐TATCGTGTGATTATCCGTGAGG‐5′), PECAM1 (forward, 5′‐AGATACTCTAGAACGGAAGG‐3′; reverse, 3′‐CAGAGGTCTTGAAATACAGG‐5′), KDR (forward, 5′‐GTACATAGTTGTCGTTGTAGG‐3′; reverse, 3′‐TCAATCCCACATTTAGTTC‐5′), and 18S (forward, 5′‐GTAACCCGTTGAACCCCATT‐3′; reverse, 3′‐CCATCCAATCGGTAGTAGGG‐5′). 18s was used as a universal housekeeping gene and fold change calculated using the delta–delta CT method.

### Endothelial Cell Tube Formation

Matrigel (Corning, 354 248)‐coated 96‐well plates were used to culture HCAECs at a concentration of 10 000 cells per well. Drugs were mixed with fresh HCAEC growth media to the listed final concentrations, and then added to the Matrigel cultures. The cells were monitored for 16 h and time‐lapse images were captured using an IncuCyte Zoom live cell imager (Essen Bioscience). To analyze the capillary network, four representative images were selected at 5 h post‐seeding from each condition with five replicate wells. The number of junctions, meshes, and segments were analyzed using the angiogenesis analyzer plugin in ImageJ.

### Silk Graft Manufacture

*Bombyx mori* silk cocoons (Tajima Shoji Co., LTD., Yokohama, Japan) were purified as previously described.^[^ ^43^ ^]^ Briefly, silk cacoons (5 g) were boiled in sodium carbonate (0.02 m, 2 L) for 30 min to remove sericin. Extracted silk fibers were washed in ultrapure water (Arium Pro, Sartorius, Göttingen, Germany) and dried overnight at room temperature before being dissolved in lithium bromide (9.3 m, LiBr) for 4 h at 60 °C to produce a 20% w/v solution. Dissolved silk–LiBr solution was transferred to SnakeSkin dialysis tubing (3500 kDa MWCO; Themo Fisher Scientific, Waltham, MA, USA) and dialyzed against 5 L of ultrapure water for 72 h. Water was changed three times a day to maintain diffusion gradient and ensure complete removal of LiBr. The solution was then centrifuged twice to remove impurities (12 000 g, 4 °C, 20 min). Silk solution was then lyophilized for 48 h and dissolved in hexafluoroisopropanol at 15% w/v.

This solution was electrospun using an IME Medical Electrospinner (IME Medical Electrospinning, Leiden, Netherlands) from a 0.6 mm diameter needle with an applied voltage of 16 kV onto a 0.5 mm stainless steel mandrel rotating at 500 rpm. Flow rate was 2 mL h^−1^, controlled by a syringe pump (Elite 11, Harvard Apparatus), and distance between needle and grounded mandrel was 220 mm. Relative humidity was regulated within the chamber and maintained at 30 ± 5%. Immediately following electrospinning, silk grafts were crosslinked on the mandrel by water annealing for 18 h.^[^ ^44^ ^]^ Residual HFP was removed by gently stirring grafts in 2 L ultrapure overnight, followed by drying at room temperature. Grafts were stored in a plastic container at room temperature with silica gel desiccant.

### Mouse Carotid Graft

Study was approved by the University of Sydney Animal Ethics Committee (protocol number 2020/1785). Experiments were conducted in accordance with the Australian Code of Practice for the Care and Use of Animals for Scientific Purpose. C57BL/6 mice (male, 9–10 weeks old, 25 ± 2 g), purchased from Animal Resources Center (Canning Vale, WA, Australia), were used for this model. Silk grafts 10 mm in length and 0.5 mm in diameter were disinfected in 70% ethanol and exposed to ultraviolet light for 30 min. The drugs were loaded by submerging silk grafts in a high concentration solution of each drug (1 mg mL^−1^ in PBS) and incubated overnight at 4 °C to ensure complete drug saturation. Drug‐coated grafts were implanted into the right common carotid artery as previously described.^[^ ^32^ ^]^ Briefly, the midpoint of the right common carotid artery was double ligated and dissected between the ligations. Polyimide cuffs (Cole–Parmer North America, Vernon Hills, Illinois) were guided onto each end of the arteries and clamped. Overhanging ends of the arteries were everted over cuffs and secured using 8–0 sutures. Silk grafts were sleeved over each cuff and secured using 8–0 sutures. Clamps were released and blood flow was confirmed with pulsation, therefore indicating successful graft implantation. After 7 or 28 days, mice were perfused using heparinized saline (50 U mL^−1^) and the grafted carotid artery was isolated and dissected proximal and distal to the graft.

### Hemocompatibility Assay

Wells in a 24 well plate were coated with 3% BSA for 1 h. Plates were then washed 3× with PBS and left to dry overnight. High porosity silk scaffold sheets, electrospun using the same properties provided above were cut into 0.8 × 1.2 cm samples, pre‐weighed and placed into individual wells. Fresh blood was collected and mixed with heparin (0.3 U mL^−1^). Blood (750 µL per well) was added onto samples followed by treatment by MCC950 (30 µm), PTX (30 µm), or SMS (30 µm). Samples were left for 1 h on an orbital shaking incubator (65 rpm, 37 °C). Blood was removed and samples were washed 3× with PBS before being weighed. Clot weight was calculated by subtracting the weight of the scaffold post‐blood incubation by initial scaffold weight.

### Histology and Immunohistochemistry

Explanted grafts were fixed in paraformaldehyde (4%) overnight at room temperature. Samples were dehydrated through an ascending ethanol gradient, embedded in paraffin and sectioned transversely at 5 µm from proximal to distal anastomosis. For histology staining, five slides from equidistant points along the graft (proximal to distal) were deparaffinized, rehydrated, and stained with H&E staining and Martius Scarlet Blue staining. The same procedure was conducted for immunohistochemistry staining but stained with primary antibodies against CD68 (1:500, ab125212, Abcam), IL‐1*β* (1:500, ab205924, Abcam), TNF‐*α* (1:200, ab34674, Abcam), CD31 (1:100, ab182981, Abcam), and eNOS (1:50, ab300071, Abcam). Secondary antibodies against rabbit (1:250, ab150080, Abcam and 1:500, ab150077, Abcam) were used to detect primary antibodies. Sections were mounted and cover slipped with DAPI‐containing mounting media (Sigma Fluoroshield with DAPI, F6057).

### Quantitative Analysis

Analysis of histology and immunohistochemistry slides was done using ImageJ. For H&E staining, neointima was quantified as percentage of total lumen area defined by the inner graft wall. For Martius Scarlet Blue staining, fibrin deposition was quantified using the “Colour Threshold” function in ImageJ to calculate amount of positive red staining present within the neointima, which was then represented as a percentage of total lumen area. Fibrotic capsule was represented as total adventitial tissue area surrounding the graft. For CD68 staining, total CD68^+^ cell count and CD68^+^ cell count within the graft wall was quantified using a constant threshold intensity. Total IL‐1*β* and TNF‐*α* positive staining area was also measured. CD31 lumen coverage was quantified by first measuring the lumen circumference, followed by measuring the length of endothelium showing positive staining. Lumen coverage was then calculated as length of CD31^+^ staining divided by lumen circumference. eNOS lumen coverage was quantified in the same way, represented as length of eNOS^+^ staining divided by lumen circumference.

### Statistical Analysis

Data are expressed as mean ± standard error of the mean. Analysis was performed in GraphPad Prism 9 (Graphpad Software, San Diego, California) and statistically significant differences were determined by *t‐*test, or using one‐ or two‐way analysis of variance followed by Dunnett's multiple comparisons test. For in vivo data, statistical analysis was performed on the grouped data comprising of all five graft regions. In vivo figures in this paper represent the respective averages of all regions for each graft. *P* < 0.05 was considered statistically significant. *, **, *** and **** display *P* < 0.05, *P* < 0.01, *P* < 0.001, and *P* < 0.0001 respectively.

## Conflict of Interest

The authors declare no conflict of interest.

## Supporting information

Supporting Information

## Data Availability

The data that support the findings of this study are available from the corresponding author upon reasonable request.
