# Supplementary material for: Selective NLRP3 Inflammasome Inhibitor MCC950 Suppresses Inflammation and Facilitates Healing in Vascular Materials
Source: Adv Sci (Weinh). 2023 May 7;10(20):2300521. doi: 10.1002/advs.202300521 (PMC10369291; doi:10.1002/advs.202300521)
Supplement: Supplementary file 1 — Supporting Information [file ADVS-10-2300521-s001.pdf]

ADVANCED SCIENCE
Open Access

Supporting Information

for *Adv. Sci.*, DOI 10.1002/advs.202300521

Selective NLRP3 Inflammasome Inhibitor MCC950 Suppresses Inflammation and Facilitates Healing in Vascular Materials

Angus J. Grant, Nianji Yang, Matthew J. Moore, Yuen Ting Lam, Praveesuda L. Michael, Alex H.P. Chan, Miguel Santos, Jelena Rnjak-Kovacina, Richard P. Tan and Steven G. Wise**

Supporting Information

The selective NLRP3 inflammasome inhibitor MCC950 suppresses inflammation and facilitates healing in vascular materials

Angus J. Grant, Nianji Yang, Mathew J. Moore, Yuen Ting Lam, Praveesuda L. Michael, Miguel Santos, Jelena Rnjak-Kovacina, Richard P. Tan[*^], Steven G. Wise[*^]

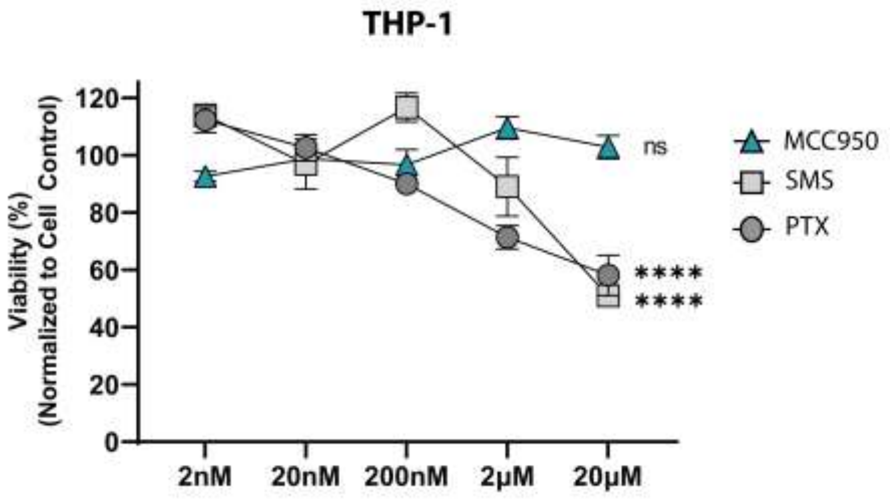

Figure S1: MCC950 is non-toxic to THP-1 derived macrophages. Viability of THP-1 derived macrophages 3 days after being treated with MCC950, Sirolimus (SMS), and Paclitaxel (PTX). Data represents mean ± SEM (n = 4). Statistical significance was determined using Dunnett's multiple comparison test ($^{****}p < 0.0001$)

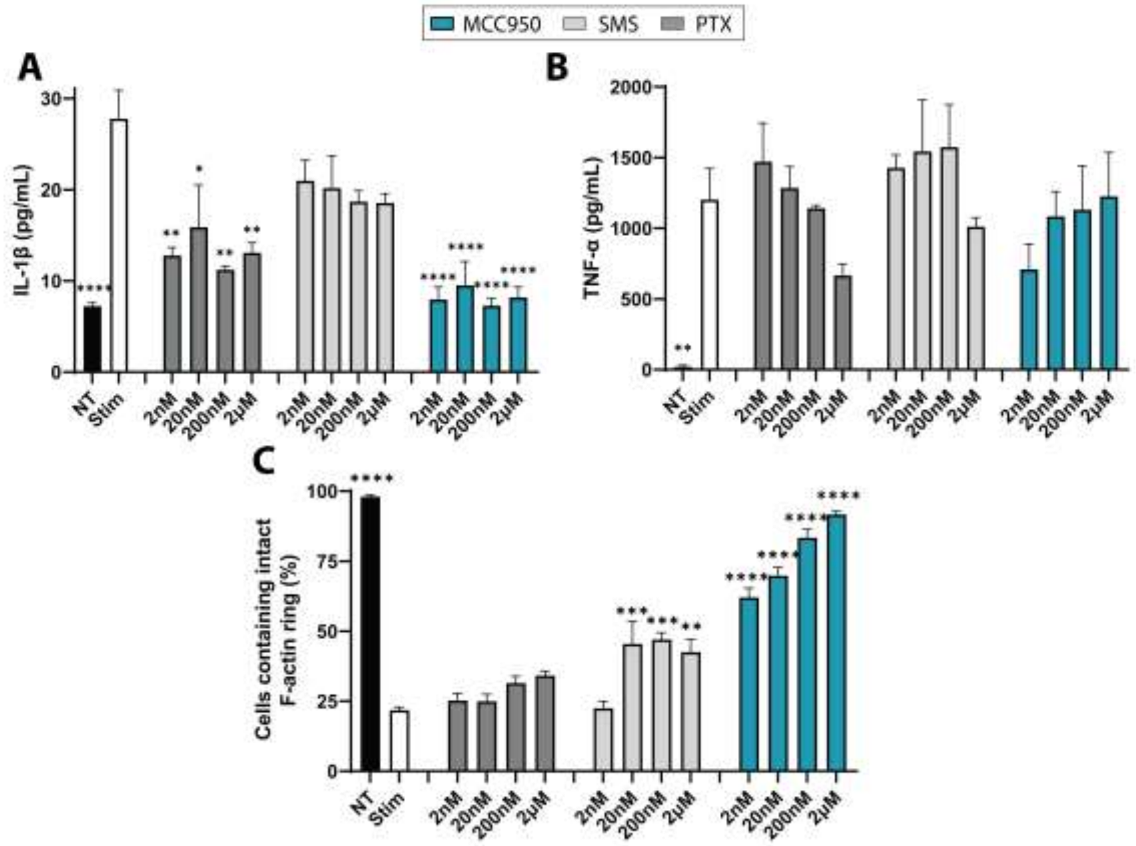

Figure S2: Inhibitory effects on products of the NLRP3 inflammasome pathway. The effect of MCC950, Paclitaxel (PTX) and Sirolimus (SMS) at doses between 2nM-2µM on IL-1β (A), TNF-α (B) and pyroptosis (C) levels post-NLRP3 inflammasome stimulation with LPS (1 µg/mL) and ATP (2.5 mM) in J774a macrophages. Stim refers to stimulated only group. IL-1β and TNF-α levels measured by ELISA. Data represents mean ± SEM (*n* = 3-4). Statistical significance was determined using Dunnett's multiple comparison test relative to stimulated group ($^{*}p < 0.05$, $^{**}p < 0.01$, $^{****}p < 0.0001$).

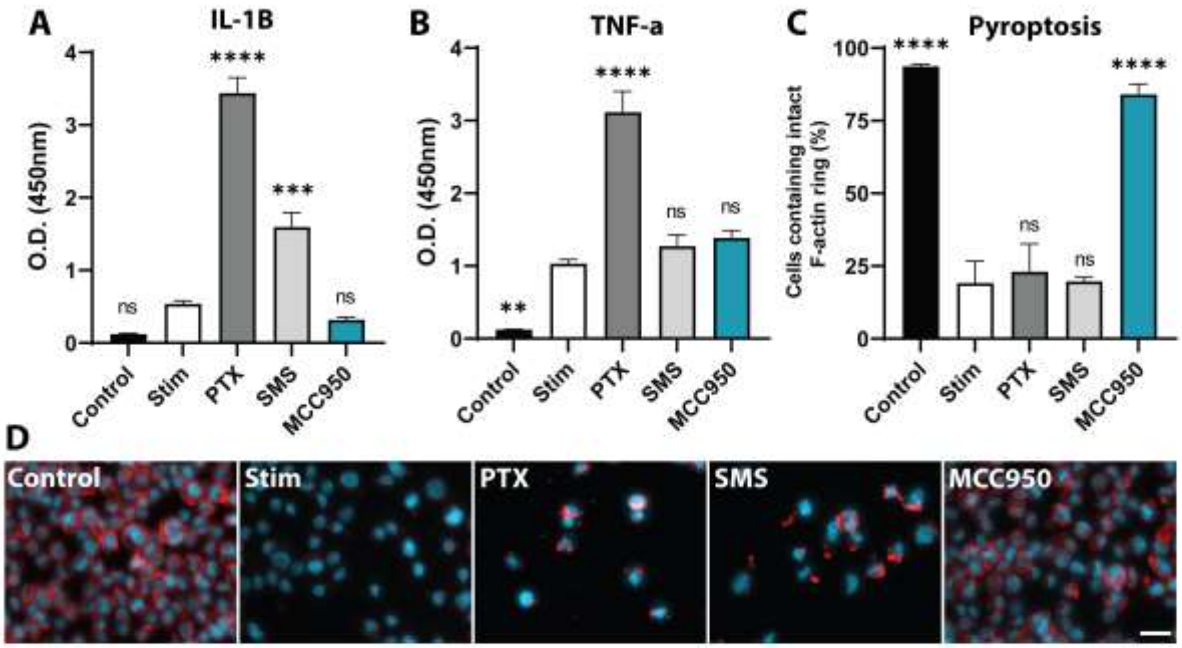

Figure S3: MCC950 is selective to the NLRP3 inflammasome pathway in THP-1 derived macrophages. The effect of high dose (20µM) MCC950, Paclitaxel (PTX) and Sirolimus (SMS) on IL-1β (A), TNF-α (B) and pyroptosis (C) levels post-NLRP3 inflammasome stimulation with LPS (1 µg/mL) and ATP (2.5 mM) in THP-1 derived macrophages. Stim refers to stimulated only group. IL-1β and TNF-α levels measured by ELISA. Data represents mean ± SEM (n = 3-4). Statistical significance was determined using Dunnett's multiple comparison test relative to stimulated group ($^{**}p < 0.01$, $^{***}p < 0.001$, $^{****}p < 0.0001$). D) Representative images of control, stimulated only, and stimulated and drug treated macrophages used for pyroptosis quantification. Cells stained with DAPI (blue) and rhodamine phalloidin (red) to visualize cell nucleus and F-actin respectively. Scale bar represents 50µm.

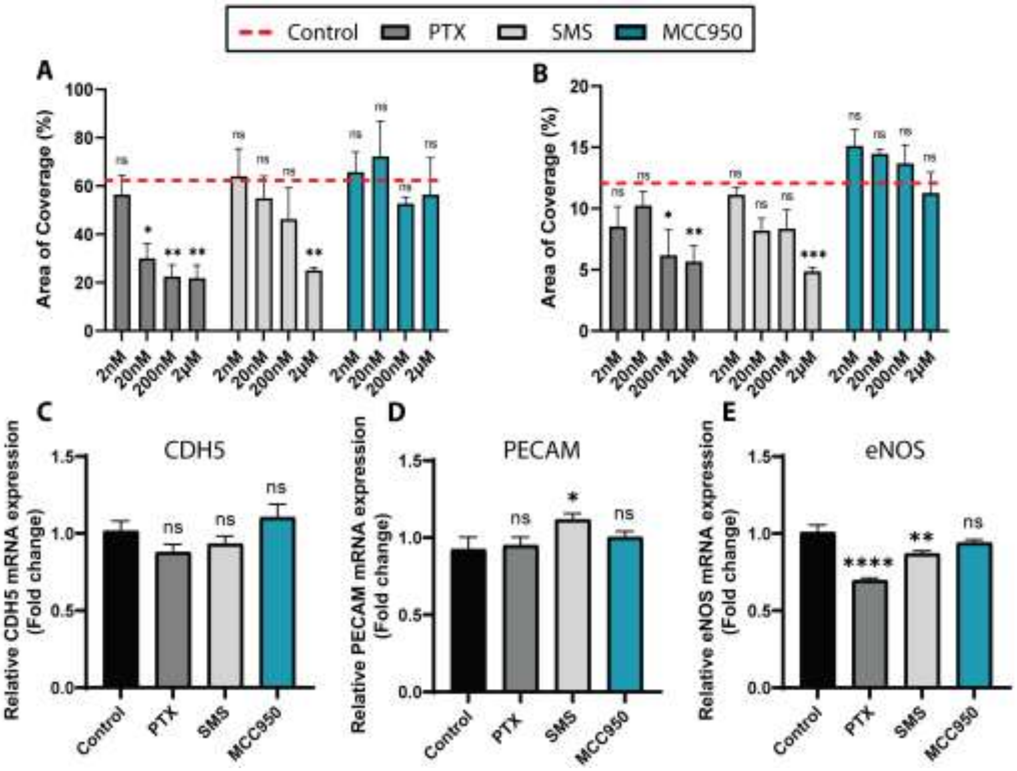

Figure S4: Endothelial functionality assay. Quantification of total HCEAEC expression of VE-Cadherin (A) and eNOS (B) 3 days after treatment with Paclitaxel (PTX), Sirolimus (SMS) or MCC950 at doses between 2nM-2μM. C, D, E) Quantitative RT-PCR detection of Cadherin 5 (CDH5) (C), platelet endothelial cell adhesion molecule (PECAM) (D), and eNOS (E) mRNA expression. Data represents mean ± SEM (*n* = 3-4). Statistical significance was determined using Dunnett's multiple comparison test relative to control ($^{*}p < 0.05$, $^{**}p < 0.01$, $^{***}p < 0.001$).

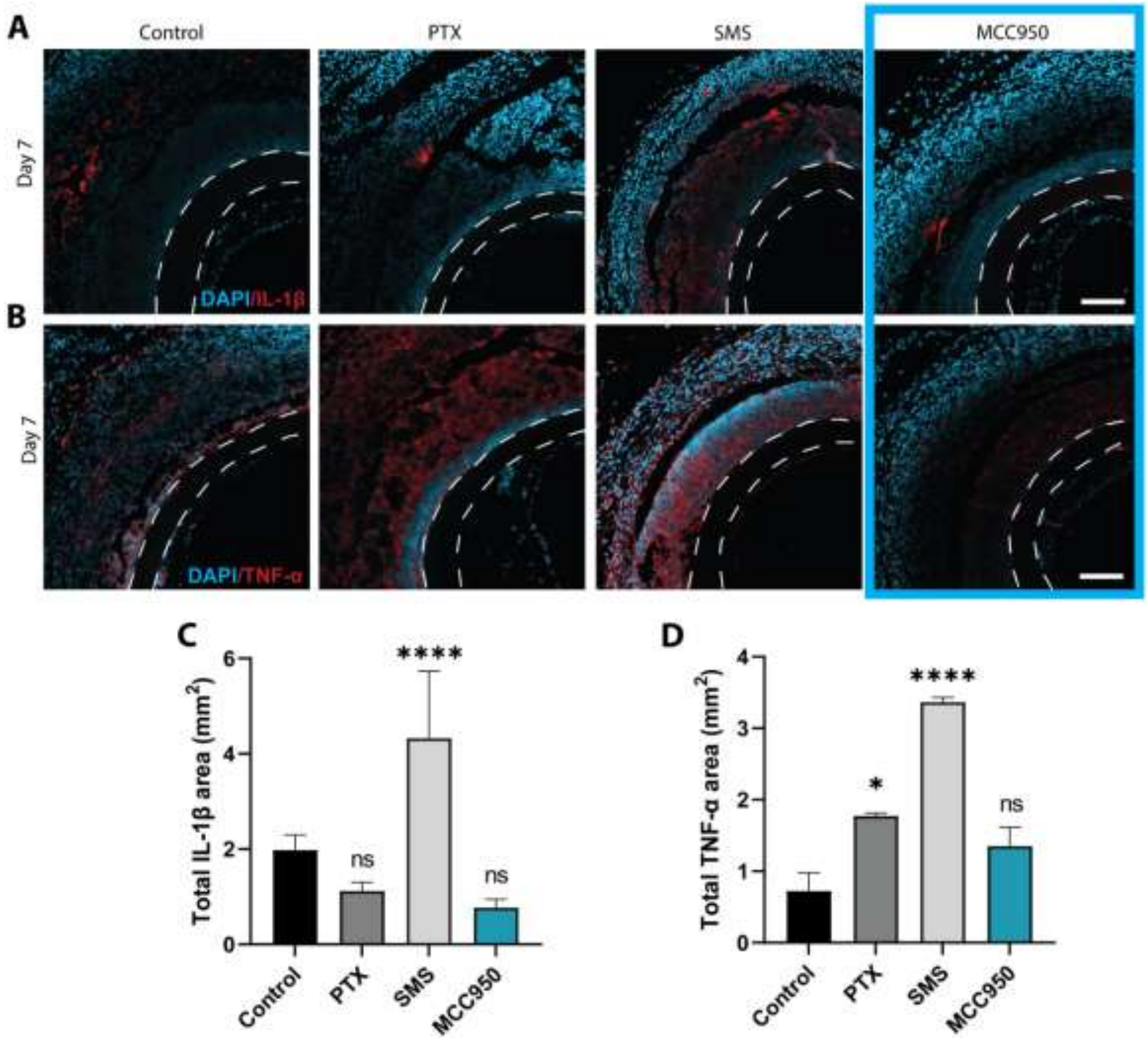

Figure S5: Treatment effect on cytokine production in vivo. A,B) Day 7 representative images of IL-1β (A) and TNF-α (B) stain taken from middle region of explanted grafts. DAPI stained in blue, IL-1β or TNF-α stained in red. Scale bar represents 100 μm. C, D) Quantification of total IL-1β (C) and TNF-α (D) positively stained area at day 7. Data represents mean ± SEM (*n* = 3-4). Statistical significance was determined using Dunnett's multiple comparison test ($^{*}p < 0.05$, $^{****}p < 0.0001$).

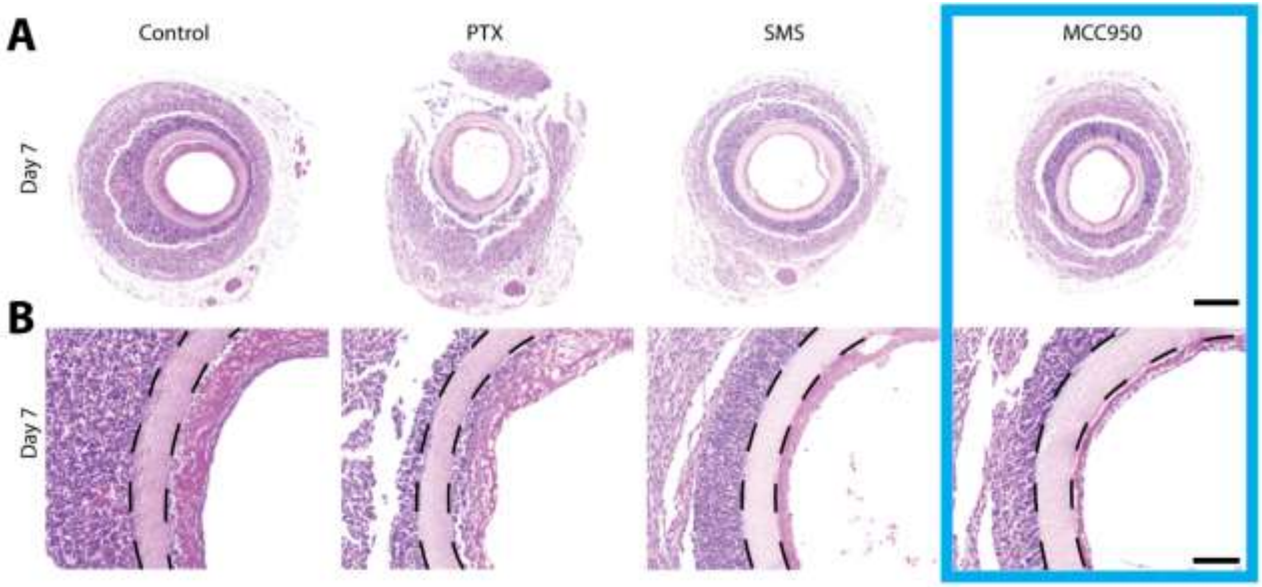

Figure S6: MCC950 reduces fibrotic capsule formation and neointimal hyperplasia. A) Representative images of hematoxylin and eosin-stained grafts after 7 days showing fibrotic capsule surrounding the graft. Images were taken from middle portion of each graft. Scale bar represents 300 µm. B) Representative images of hematoxylin and eosin-stained grafts after 7 days showing neointimal hyperplasia. Black dotted lines show graft outline. Scale bar represents 100 µm.
